# A multicenter evaluation of a deep learning software (LungQuant) for lung parenchyma characterization in COVID-19 pneumonia

**DOI:** 10.1186/s41747-023-00334-z

**Published:** 2023-04-10

**Authors:** Camilla Scapicchio, Andrea Chincarini, Elena Ballante, Luca Berta, Eleonora Bicci, Chandra Bortolotto, Francesca Brero, Raffaella Fiamma Cabini, Giuseppe Cristofalo, Salvatore Claudio Fanni, Maria Evelina Fantacci, Silvia Figini, Massimo Galia, Pietro Gemma, Emanuele Grassedonio, Alessandro Lascialfari, Cristina Lenardi, Alice Lionetti, Francesca Lizzi, Maurizio Marrale, Massimo Midiri, Cosimo Nardi, Piernicola Oliva, Noemi Perillo, Ian Postuma, Lorenzo Preda, Vieri Rastrelli, Francesco Rizzetto, Nicola Spina, Cinzia Talamonti, Alberto Torresin, Angelo Vanzulli, Federica Volpi, Emanuele Neri, Alessandra Retico

**Affiliations:** 1 Physics Department, University of Pisa, Pisa, Italy; 2 Pisa Division, National Institute for Nuclear Physics, Pisa, Italy; 3 Genova Division, National Institute for Nuclear Physics, Genova, Italy; 4 Department of Political and Social Sciences, University of Pavia, Pavia, Italy; 5 Pavia Division, National Institute for Nuclear Physics, Pavia, Italy; 6 Department of Medical Physics, ASST Grande Ospedale Metropolitano Niguarda, Milan, Italy; 7 Milano Division, National Institute for Nuclear Physics, Milan, Italy; 8 Department of Experimental and Clinical Biomedical Sciences, Radiodiagnostic Unit n. 2, University of Florence-Azienda Ospedaliero-Universitaria Careggi, Florence, Italy; 9 Unit of Imaging and Radiotherapy, Department of Clinical-Surgical, Diagnostic and Pediatric Sciences, University of Pavia, Pavia, Italy; 10 Institute of Radiology, Department of Diagnostic and Imaging Services, Fondazione IRCCS Policlinico San Matteo, Pavia, Italy; 11 Department of Mathematics, University of Pavia, Pavia, Italy; 12 Department of Biomedicine, Neuroscience and Advanced Diagnostic (BiND), University of Palermo, Palermo, Italy; 13 Department of Translational Research, Academic Radiology, University of Pisa, Pisa, Italy; 14 Post-graduate School in Radiodiagnostics, University of Milan, Milan, Italy; 15 Department of Physics “Aldo Pontremoli”, University of Milan, Milan, Italy; 16 Department of Physics and Chemistry “Emilio Segrè”, University of Palermo, Palermo, Italy; 17 Catania Division, National Institute for Nuclear Physics, Catania, Italy; 18 Cagliari Division, National Institute for Nuclear Physics, Monserrato, Cagliari, Italy; 19 Department of Chemical, Physical, Mathematical and Natural Sciences, University of Sassari, Sassari, Italy; 20 Department of Radiology, ASST Grande Ospedale Metropolitano Niguarda, Milan, Italy; 21 Postgraduate School of Diagnostic and Interventional Radiology, University of Milan, Milan, Italy; 22 Department Biomedical Experimental and Clinical Science “Mario Serio”, University of Florence, Florence, Italy; 23 Florence Division, National Institute for Nuclear Physics, Sesto Fiorentino, Firenze, Italy; 24 Department of Oncology and Hemato-Oncology, University of Milan, Milan, Italy; 25 Italian Society of Medical and Interventional Radiology, SIRM Foundation, Milan, Italy

**Keywords:** COVID-19, Deep Learning, Lung, Software validation, Tomography (x-ray computed)

## Abstract

**Background:**

The role of computed tomography (CT) in the diagnosis and characterization of coronavirus disease 2019 (COVID-19) pneumonia has been widely recognized. We evaluated the performance of a software for quantitative analysis of chest CT, the *LungQuant* system, by comparing its results with independent visual evaluations by a group of 14 clinical experts. The aim of this work is to evaluate the ability of the automated tool to extract quantitative information from lung CT, relevant for the design of a diagnosis support model.

**Methods:**

*LungQuant* segments both the lungs and lesions associated with COVID-19 pneumonia (ground-glass opacities and consolidations) and computes derived quantities corresponding to qualitative characteristics used to clinically assess COVID-19 lesions. The comparison was carried out on 120 publicly available CT scans of patients affected by COVID-19 pneumonia. Scans were scored for four qualitative metrics: percentage of lung involvement, type of lesion, and two disease distribution scores. We evaluated the agreement between the *LungQuant* output and the visual assessments through receiver operating characteristics area under the curve (AUC) analysis and by fitting a nonlinear regression model.

**Results:**

Despite the rather large heterogeneity in the qualitative labels assigned by the clinical experts for each metric, we found good agreement on the metrics compared to the *LungQuant* output. The AUC values obtained for the four qualitative metrics were 0.98, 0.85, 0.90, and 0.81.

**Conclusions:**

Visual clinical evaluation could be complemented and supported by computer-aided quantification, whose values match the average evaluation of several independent clinical experts.

**Key points:**

- We conducted a multicenter evaluation of the deep learning-based *LungQuant* automated software.
- We translated qualitative assessments into quantifiable metrics to characterize coronavirus disease 2019 (COVID-19) pneumonia lesions.
- Comparing the software output to the clinical evaluations, results were satisfactory despite heterogeneity of the clinical evaluations.
- An automatic quantification tool may contribute to improve the clinical workflow of COVID-19 pneumonia.

**Supplementary Information:**

The online version contains supplementary material available at 10.1186/s41747-023-00334-z.

## Background

The role of computed tomography (CT) in the diagnosis and characterization of coronavirus disease 2019 (COVID-19) pneumonia is widely recognized [1–4]. The main manifestations of COVID-19 pneumonia on chest CT are summarized in various reports [5, 6], and combined qualitative and quantitative indicators of lung CT have been shown to be useful for the assessment of the severity of the disease [7, 8]. Preliminary identification of COVID-19 lung lesions is needed to evaluate these indicators. Deep learning (DL) has the potential to help in this task, and various DL-based tools have been developed to address COVID-19 lesions on CT scans [9–12].

Typical validation studies for segmentation software are based on a direct comparison of the software output (*i.e.*, segmented region of interest masks of organs or lesions) with the manual segmentation traced by radiologists, the latter being considered the gold standard [13, 14]. However, very few studies compared the clinical assessment of CT describing the type of lesion and its spatial distribution with the output of a software. In [15], the authors compared the quantitative and qualitative CT parameters obtained visually and by software in COVID-19 disease, with the aim to understand which parameters are predictors of mortality. In [16], they compared the performance of an automated DL-based method to manual scoring the lung involvement on chest CT scans to find the optimal threshold for quantification of the affected lung region in COVID-19 pneumonia.

We used an automated DL-based segmentation and quantification tool, called *LungQuant* [17]. It has been already statistically validated for its segmentation performance on multiple datasets as already described [17], *i.e.*, by means of a standard partitioning of the dataset into training, validation, and test sets and using as metric the surface and volumetric Dice similarity coefficients, which are the most commonly used metrics to evaluate an artificial intelligence (AI) segmentation algorithm.

The aim of the present work was to evaluate the ability of the software to provide descriptive metrics that can support clinicians in the characterization of lesions detected on CT scans of patients affected by COVID-19 pneumonia. Therefore, our focus was on the ability of the software to translate qualitative metrics into quantifiable values, and we validated this by means of a statistical comparison to multicenter clinical assessments.

## Methods

We selected four qualitative parameters evaluated on lung CT scans that provide prognostic information on COVID-19: CT severity score (CTSS, 5 grades); lesion type (5 categories); bilateral involvement (yes/no); and basal predominance (yes/no). A subjective visual assessment of these qualitative clinical parameters was independently collected from 14 radiologists operating in 5 clinical centers. Given the large number of independent clinical evaluators, we set the reference standard to be a statistical measure over all independent clinical outputs. In addition, starting from the region of interest of lesion segmentation delivered by the *LungQuant* software [17], we computed four continuous metrics to match the corresponding qualitative parameters describing the COVID-19 lesions. We then used these derived metrics to fit the visual assessments using a logistic regression model.

The *LungQuant* system [17] is a DL-based analysis pipeline for the identification, segmentation, and quantification of COVID-19 pulmonary lesions, already described and validated according to the CLAIM checklist [18]. In this study, we used an updated version of the algorithm [17], publicly available in an open access repository (https://www.openaccessrepository.it/record/76937#.Y_dQhjZKgUE), based on a cascade structure of neural network architectures, CNN and U-nets. Briefly, one CNN is trained to predict a bounding box that surrounds the lungs, while the U-nets are devoted to segmenting the lungs and lesions. The software outputs:

- The lung parenchyma segmentation mask;
- The COVID-19 lesion segmentation mask, including ground-glass opacities (GG) and consolidations (Co);
- The percentage of lung volume affected by COVID-19 lesions;
- And the CTSS, defined as score 1 for percentage *<* 5%, score 2 for 5% *≤*percentage *<* 25%; score 3 for 25% *≤*percentage *<* 50%, score 4 for 50% *≤* percentage *<* 75%, and score 5 for percentage *≥* 75%.

**Fig. 1:**
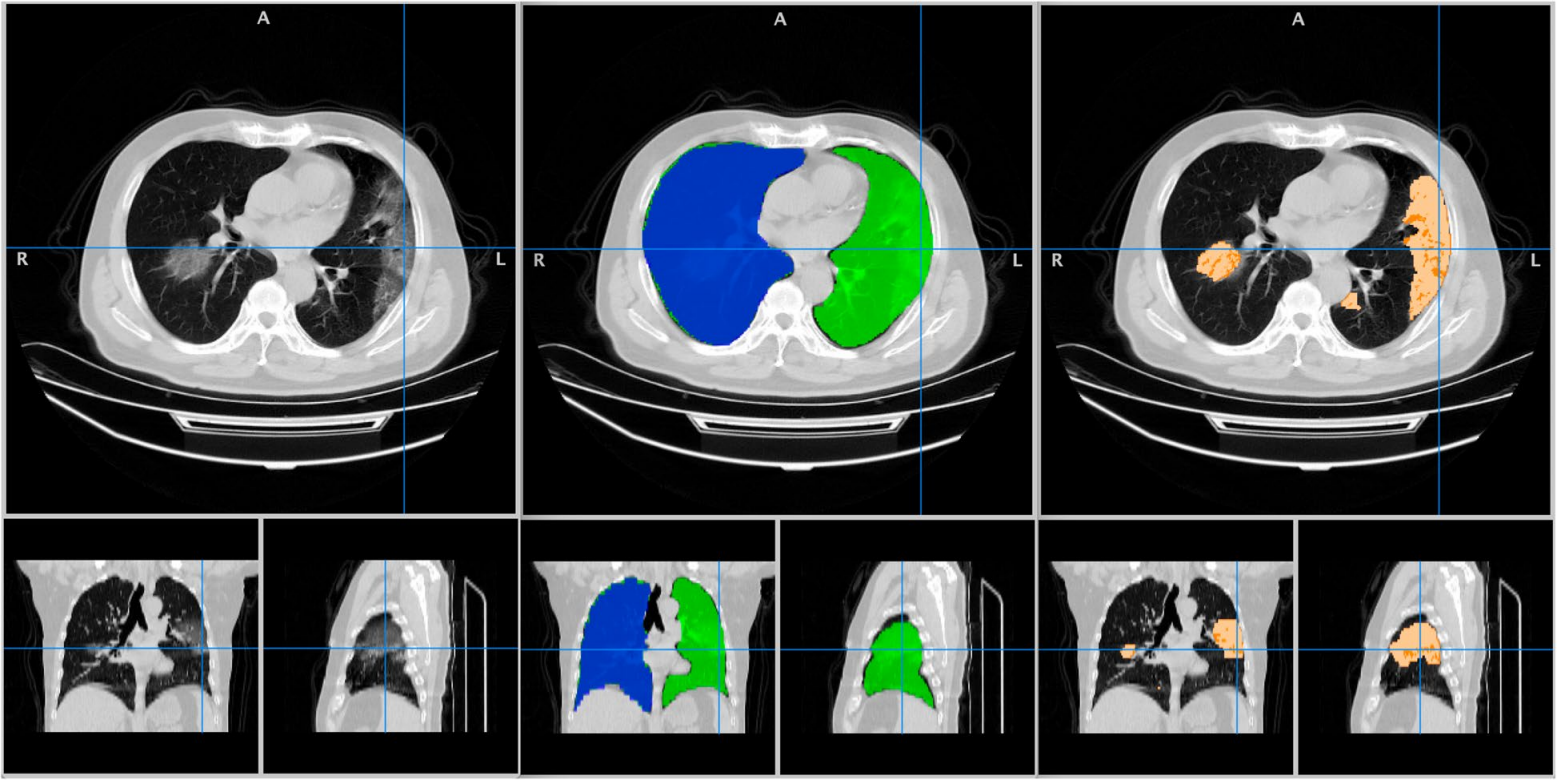
The output of *LungQuant* for a lung computed tomography scan. Left: original input image with axial, coronal and sagittal projections. Center: lung mask produced by *LungQuant* with different labels for the right and left lungs. Right: lesion masks produced by *LungQuant* for the ground-glass opacities (light orange) and the consolidations (dark orange)

Figure 1 shows an example of *LungQuant* output. Further details on *LungQuant* are provided in the Supplementary Materials.

### Dataset

After a review of the public COVID-19 datasets available for lung CT scans [19], we selected 120 CT scans to use for visual evaluation and comparison with the software. Scans were sampled from *The Cancer Imaging Archive* public database [20], which includes only patients with severe acute respiratory syndrome coronavirus-2 (SARS-CoV-2) infection confirmed by reverse transcription polymerase chain reaction. The 120 scans were randomly selected so that they were not used in the training of *LungQuant* [21], but were sampled with a CTSS statistics similar to the one used for the training of the software. The number of 120 scans was chosen to not overload clinicians. Images were available to download in the NIfTI file format and were fully anonymized. The original image intensity in Hounsfield units and the voxel size were the only information available on the scan (Table 1), while the acquisition parameters or patient information were not provided.

**Table 1:** Distribution of the 120 cases for two available parameters, slice thickness and arm position

Slice thickness (mm)	Number of cases	Arms position	Number of cases
5	114	up	110
2	2	down	10
1	4		

Arm position was defined as the raising (up) or not (down) of the arms above the head

All CT scans and their related *LungQuant* segmentations have been visually inspected to screen for acquisition issues, subpar signal-to-noise ratio, and processing faults. No scans were rejected.

### Qualitative evaluation metrics

First, we collected from each of the 14 radiologists who participated in the project the following self-assessed indicators of expertise (Table 2):

- Thoracic CT experience (rough estimation of the knowledge of thoracic radiology expressed in years: *<* 5 years, 5*−*10 years, *>* 10 years);
- COVID-19 CT expertise (rough estimation of the expertise to evaluate CT COVID-19 cases expressed in the number of cases: *<* 100 cases, 100*−*400 cases, *>* 400 cases;
- Self-confidence: a subjective indication of confidence in evaluating CT scans, ranging from 1 (very low) to 5 (very high).

**Table 2:** Summary statistics of the readers’ self-assessment of their expertise

Experience	Levels	Number of radiologists (%)
Thoracic CT experience (years)	< 5	10 (72%)
	5−10	1 (7%)
	> 10	3 (21%)
COVID-19 expertise (number of cases)	< 100	3 (21%)
	100−400	8 (58%)
	> 400	3 (21%)
Self-confidence	1	0 (0%)
	2	4 (29%)
	3	4 (28%)
	4	4 (29%)
	5	2 (14%)

*COVID-19* Coronavirus disease 2019, *CT* Computed tomography

**Fig. 2:**
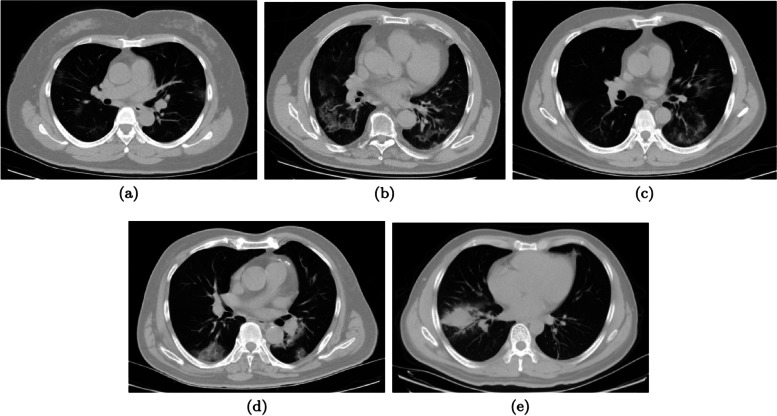
Lesion type examples (axial projection only). **a** Ground-glass only (patient ID: A0037). **b** Mainly ground-glass opacities (patient ID: A0311). **c** Similar contribution of ground-glass and consolidations (patient ID: A0266). **d** Mainly consolidations (patient ID: A0327). **e** Consolidations only (patient ID: A0509)

**Fig. 3:**
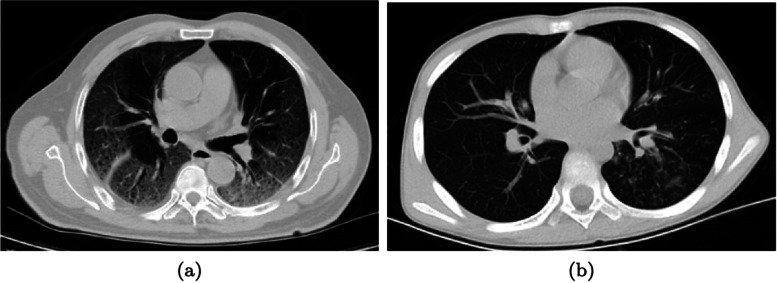
Distribution of the lesions. **a** An example with a bilateral distribution of the lesions (patient ID: A0028 1). **b** An example without bilateral distribution where the lesions are present in one lung only (patient ID: A0684)

**Fig. 4:**
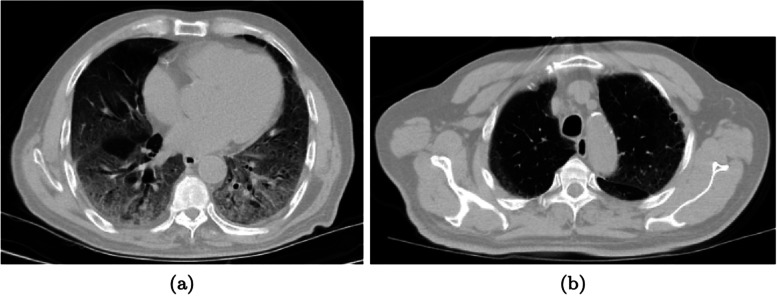
Distribution of the lesions. An example with a basal predominant distribution. Two representative slices in the axial projection of the same patient (patient ID: A0028 1): bases with lesions involvement (**a**); apices free of lesions (**b**). A coronal or a sagittal projection would have better illustrated the gradient, but the quality of these projections was worse because of the large slice thickness

The following metrics have been identified by the authors prior to the study and on the basis of common clinical knowledge, to be relevant both to characterize COVID-19 pneumonia and to be common practice in the routine evaluation of chest CT scans:

- lesion type, to be selected among:
  - GGO (ground glass only): only GG opacities are present (Fig. 2a). GG has been reported as the primary finding of COVID-19 pneumonia on CT scans [21]. It appears as a hazy increase in opacity of the lungs, with preservation of the bronchial and vascular margins [22].
  - MGG (mainly ground glass): most of the lesion is GG, but scattered consolidation sites are also present (Fig. 2b).
  - CoGG (consolidation and GG): GG and consolidations are present in approximately similar proportions (Fig. 2c).
  - MCo (mainly consolidations): most of the lesion is consolidation, but GG is also visible (Fig. 2d).
  - CoO (consolidations only): only consolidations are present (Fig. 2e). They appear as a homogeneous increase in pulmonary parenchymal attenuation that obscures the margins of the vessels and airway walls [23], typically associated with a more severe prognosis [6].
  No thresholds were established to discriminate among the types of lesion; decision-making was left to each radiologist.
- Lesion distribution, with each of the following properties described by a binary label (yes/no):
  - Bilateral, when pulmonary lesions are visible in both lungs in an approximately similar percentage (Fig. 3) [24];
  - Basal predominant: lesions affect mainly the bases of the lungs with relative sparing of the upper regions (Fig. 4) [4].

In addition, these qualitative assessments were defined:

- Image quality, to be selected among “optimal”, “acceptable”, and “low”, without formal definition of quality (typically, suboptimal quality involves image acquisition flaws, such as field of view cuts and artifacts from patient movement or metal materials, presence of excessive noise, *etc.*). While this label was assigned according to the reader’s own experience, all 14 readers were familiar with standard clinical protocols for thoracic CT acquisition/reconstruction; therefore we assumed homogeneous evaluation [25] (Table 2);
- Clinical CTSS, *i.e.*, estimate of the compromised lung fraction by assigning a 5-class score (1 = 0−5%; 2 = 5–25%; 3 = 25−50%; 4 = 50−75%; and 5 = 75−100%).

### Visual evaluation protocol

The 120 scans were independently presented to 14 radiologists from 5 Italian clinical centers: the Universities of Florence, Milan, Palermo, Pavia, and Pisa.

In order to avoid work overload, the 120 cases were divided into 3 batches of 40 cases. The evaluation of each batch was completed sequentially within 2 weeks from the start of the assignment and there was an interval of 1 week between batches. The anonymized scans were presented in native space and randomized, and the readers were blind to the output of the other colleagues.

Each reader was free to use the most appropriate visualization tool to navigate and examine the image volume. No indication was given on the most appropriate windowing and image parameters, so the evaluations were carried out according to each reader’s own expertise.

This evaluation protocol does not seek consensus and each reader was asked to provide an independent opinion. This allowed us to consider each reader’s assessment as unrelated to that of the others. For each scan, we received 14 independent evaluations (for a total of 14 × 120 = 1,680 instances) and, based on the statistical hypothesis of independence, we postulated that the expected value of their assessments is the unbiased estimator of the ground truth, which we considered the gold standard.

### *LungQuant* output

The 120 cases were also analyzed by *LungQuant*, whose output consists of the lung/lesion segmentation masks and a set of volumetric estimates computed on the masks. Raw *LungQuant* outputs were:

- *Lung_volume*, total volume of the lungs;
- *LL_ratio*, the ratio between the total volume of the lesion and the total volume of the lungs;
- *consolidation_volume*, volume of consolidations in the lesion mask;
- *lesion_ volume*, total volume (right + left) of the lesion (GG + consolidations);
- *R_gg*, volume of GG in the right lung;
- *L_gg*, volume of GG in the left lung;
- *L_con*, volume of consolidations in the left lung;
- *R_con*, volume of consolidations in the right lung.

From these raw values, we computed the corresponding metrics to match the radiologist evaluations:

- *Lesion Type*_*LQ*_, defined as

1 $${\textit{Lesion}\mathit\;\textit{Type}}_\textit{LQ}=\frac{\textit{consolidation}\mathit\;\textit{volume}}{\textit{lesion}\mathit\;\textit{volume}}$$

when this index is closer to zero, the lesion is mainly GG; when it is closer to 1, the lesion is mostly consolidation;

- Bilateral_LQ_, defined as

2 $${Bilateral}_{LQ}=1-\frac{\mid\left(R_\text{con}+R_\text{gg}\right)-\left(L_\text{con}+L_\text{gg}\right)\mid}{\textit{lesion}\mathit\;\textit{volume}}$$

the lower the index, the less bilateral the lesion;

- *Basal*_*LQ*_, obtained by projecting both the lung distribution and the lesion distribution on the z-axis (the lung axis). The index value is calculated as the percentile of the lung distribution which lies the median of the lesion distribution. A lower index corresponds to a lower z and therefore to a more basal distribution of the lesions.

### Statistical analysis

#### Agreement measures

The concordance of the readers was evaluated with the intraclass correlation coefficient (ICC), accuracy, and Cohen’s κ for the CTSS, the lesion type, and lesion distribution. ICC measures are agreement, consistency, and reliability, both with single and mixed formulations. The interpretation was ICC *≤* 0*.*50 = poor; 0*.*50 ICC *≤* 0*.*75 = moderate; 0*.*75 ICC *≤* 0*.*90 = good; and ICC *≥* 0*.*90 = excellent [26]. The analysis was stratified by clinical experience on COVID-19 CT scans.

A further measure of agreement (labeled readers’ *accord*) was defined on each scan as follows: the fraction of readers sharing the same opinion on a clinical metric (CTSS, lesion type, lesion distribution). For example, suppose we test for GGO on a given CT scan, then we had *accord* 1 when either all readers agreed on the GGO evaluation, or all readers exclude the GGO evaluation. So, *accord* 1 was obtained in the case of full agreement on the presence/absence of a CT characteristic. On the other end, *accord* 0 meant an undecided case, *i.e.*, half of the readers have opinion “*x*” the other half have opinion “not-x”. In the example, half of the readers assigned the GGO lesion type while the other half assigned another label (MGG, CoCC, MCo, or CoO). The accord values are useful to test whether a characteristic is apparent and well understood by the clinicians. We computed the cumulative fraction of cases as a function of the accord (*i.e.*, the number of cases over the 120 total with accord less than a given threshold).

#### AUC analysis and sigmoid model

The link between the *LungQuant* output and the reader’s evaluation was quantified by receiver operating characteristics area under the curve (AUC) analysis and non-linear regression.

With the AUC analysis, we evaluated the discrimination ability of the *LungQuant* outputs with respect to the “true” clinical evaluations. Given the assumption of independence, the “true” evaluation was estimated as the sample mean on the clinicians’ opinions. For binary labels (yes/no), the sample mean is simply the fraction of readers who reported “yes”, while for a multilevel evaluation a strict rank among the levels must be assumed. In the case of lesion types, the rank was assumed to be as follows: GGO *<* MGG *<* CoGG *<* MCo *<* CoO, in par with the lesion gravity. Similarly, the CTSS was naturally ranked into a progressively higher lesion-to-lung ratio. The rank was then translated into natural numbers 1 *... n* to compute the necessary statistics.

Therefore, each scan was assigned its “true” evaluation estimate and these values were compared to the corresponding *LungQuant* metrics. For the evaluation of the lesion type, we defined four ranges: [GGO, MGG), [MGG, CoGG), [CoGG, MCo) and [MCo, CoO]. In the binary case, the negative/positive scans are those whose sample mean is *<* 0*.*5 (prevalence of “no”)/*≥* 0*.*5 (prevalence of “yes”) respectively, while in the case of lesion type the negative population was defined as those scans whose sample mean belonged to [GGO, CoGG) and the positive population to the complementary set [CoGG, CoO]. Similarly, for the CTSS we used [0, 50] and [50, 100] as contrasting populations. The cutoff values were calculated using the Youden index.

The sigmoid model is a common approach in nonlinear regression analysis to link a continuous variable to a set of ordinal classes (see [27] for a similar analysis applied to nuclear medicine neuroimaging). In the simplest declination of a 2-levels set, it reduces to the logistic regression, while with a multi-level set we used the same ordinal rank as in the AUC computation. For added robustness and comparison, the sigmoid regression was computed using two methods: one based on the continuation of the linear regression—so that the sigmoid slope is equal to the linear slope—and another based on the unrestricted, nonlinear regression of the sigmoid function.

The sigmoid model naturally delivers the inflection point and the slope as characteristic parameters to compare the cutoff and sample stratifications. These parameters and *LungQuant* discrimination ability (AUC values and Youden cutoff points) are reported as a function of COVID-19 and thoracic CT expertise.

## Results

### Agreement measures

**Fig. 5:**
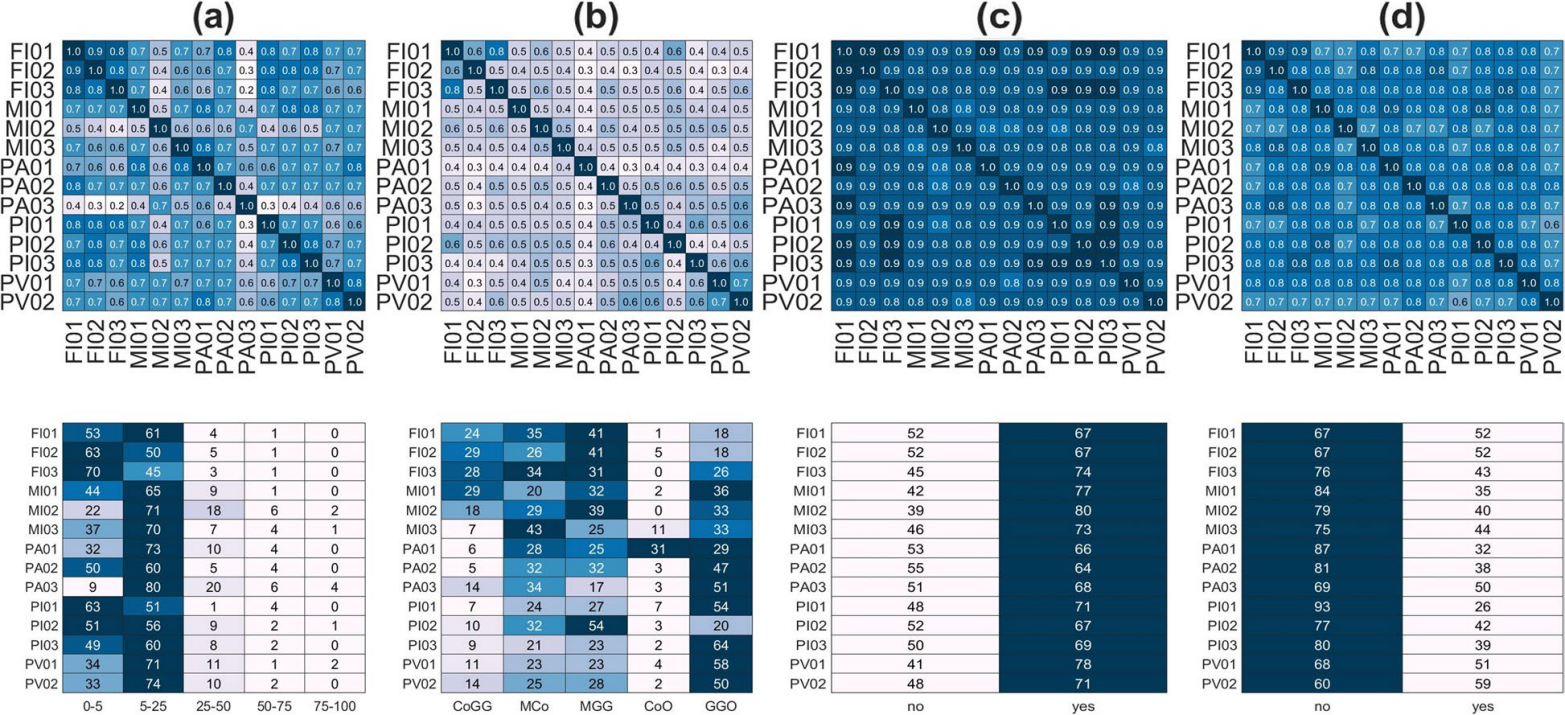
Top row: accuracy matrix on the four clinical metrics: computed tomography severity score (**a**), lesion type (**b**), bilateral (**c**), and basal predominant (**d**) lesion distribution. Bottom row: number of cases per reader and metric class

The readers’ agreement is shown in Fig. 5 for all clinical metrics. Accuracy values are given for all reader pairs. In the same figure, we report the number of cases per lesion class and reader.

The average intrarater accuracy calculated on the off-diagonal elements on the CTSS and lesion type is rather poor (mean *±* standard deviation): CTSS accuracy 0*.*65 *±* 0*.*13 (Cohen’s κ 0*.*41 *±* 0*.*19); lesion type accuracy 0*.*47 *±* 0*.*08 (Cohen’s κ 0*.*30 *±* 0*.*09). The stratification with COVID-19 expertise does not show significant trend with the expertise (*t* test *p* = 0.200): lesion type accuracy 0*.*49 *±* 0*.*07 for readers sharing high expertise (#*n >* 400) and 0*.*42 *±* 0*.*03 for readers with relatively low expertise (#*n <* 100).

The global ICC values (agreement, consistency, and reliability) are shown in Table 3 where we also stratified the values for the COVID-19 experience. Accuracy tables for all metrics stratified by COVID-19 experience are reported in the Supplementary Materials.

**Table 3:** Intraclass correlation coefficient values for the clinical metrics computed for all readers and stratified by COVID-19 expertise and scan quality

Metric	# Rad	owaA	raaA	twrssC	twrmaC	twrssR	twrmaR	Sample	Strata
CTSS	14	0.63	0.96	0.70	0.97	0.63	0.96	120	All
	3	0.63	0.84	0.72	0.88	0.64	0.84	120	> 400
	3	0.64	0.84	0.68	0.86	0.65	0.85	120	< 100
	8	0.60	0.92	0.69	0.95	0.61	0.93	120	100−400
	14	0.69	0.97	0.75	0.98	0.69	0.97	83	*>*Acceptable
Lesion type	14	0.45	0.92	0.49	0.93	0.45	0.92	120	All
	3	0.41	0.68	0.49	0.75	0.44	0.70	120	> 400
	3	0.38	0.65	0.48	0.73	0.41	0.67	120	< 100
	8	0.46	0.87	0.48	0.88	0.46	0.87	120	100−400
	14	0.43	0.91	0.47	0.93	0.43	0.91	83	*>*Acceptable
Bilateral	14	0.76	0.98	0.76	0.98	0.76	0.98	120	All
	3	0.75	0.90	0.76	0.91	0.75	0.90	120	> 400
	3	0.73	0.89	0.73	0.89	0.73	0.89	120	< 100
	8	0.77	0.96	0.77	0.96	0.77	0.96	120	100−400
	14	0.79	0.98	0.80	0.98	0.79	0.98	83	*>*Acceptable
Basal predominant	14	0.52	0.94	0.53	0.94	0.52	0.94	120	All
	3	0.53	0.77	0.54	0.78	0.53	0.77	120	> 400
	3	0.60	0.82	0.61	0.82	0.60	0.82	120	< 100
	8	0.48	0.88	0.50	0.89	0.49	0.88	120	100−400
	14	0.53	0.94	0.54	0.94	0.53	0.94	83	*>*Acceptable

Scans being labeled at least once as “low quality” were excluded

*# Rad* Number of radiologists in the stratum, *Sample* Number of scans in the stratum, *owaA* One-way absolute agreement, *raaA* Random-average absolute agreement, *twrssC* Two-way-random single-score consistency, *twrmaC* Two-way-random mixed-average consistency, *twrssR* Two-way-random single-score reliability, *twrmaR* Two-way-random mixed-average reliability

**Fig. 6:**
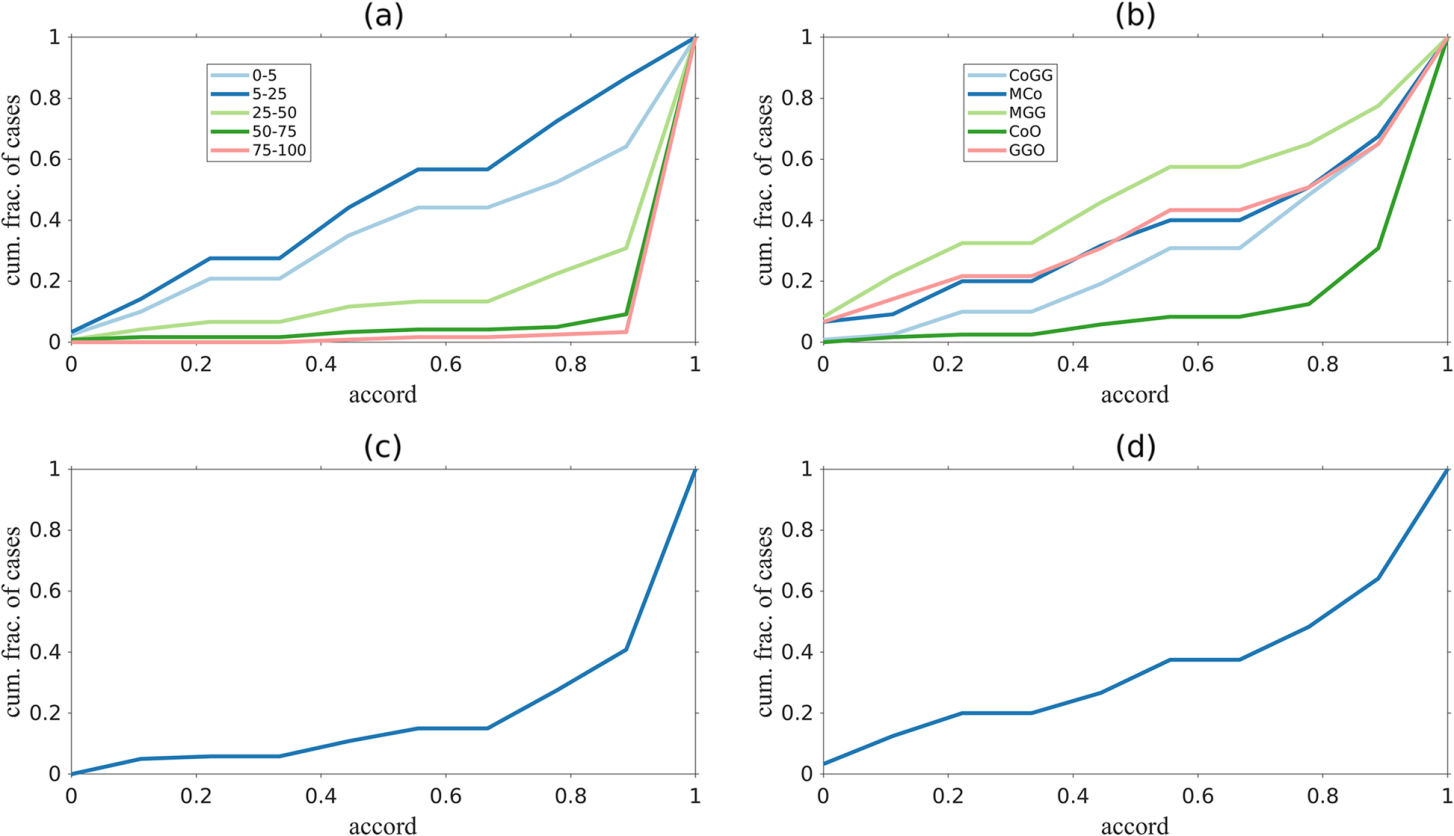
Cumulative fraction of cases as function of the accord for all clinical metrics: computed tomography severity score (**a**), lesion type (**b**), bilateral (**c**), and basal predominant (**d**) lesion distribution. Curves show the fraction of cases with accord less or equal to a given value. The larger the area under the curves, the lower the general accord. An accord = 1 indicates complete agreement, an accord equal to 0 indicates that half of the evaluators shared the opinion “x” (where “x” is one of the possible choices for a given metric), the other half shared the opinion “not-x”

Figure 6 shows the cumulative fraction of cases as a function of the accord. As a figure of merit, we indicate the cumulative fraction of cases with *accord ≤* 0*.*5 (*i.e.*, 75% of the readers share the same opinion). Clinical metrics have distinctive profiles, for example: scans with high CTSS are evaluated with higher concordance than those with low CTSS (only 4% and 1% of CTSS with involvement *>* 50% and *>* 75% respectively were evaluated with an *accord ≤* 0*.*5). Similarly, the cumulative fraction of cases with *accord* 0.5 on the lesion type were 0.25, 0.36, 0.52, 0.07, and 0.37 for CoGG, MCo, MGG, CoO, and GGO, respectively.

Finally, for the distribution of the lesions, the evaluation of the basal characteristic is less consistent than the bilateral evaluation (0.32 *versus* 0.13 at *accord* 0.5).

### AUC analysis and sigmoid model

**Fig. 7:**
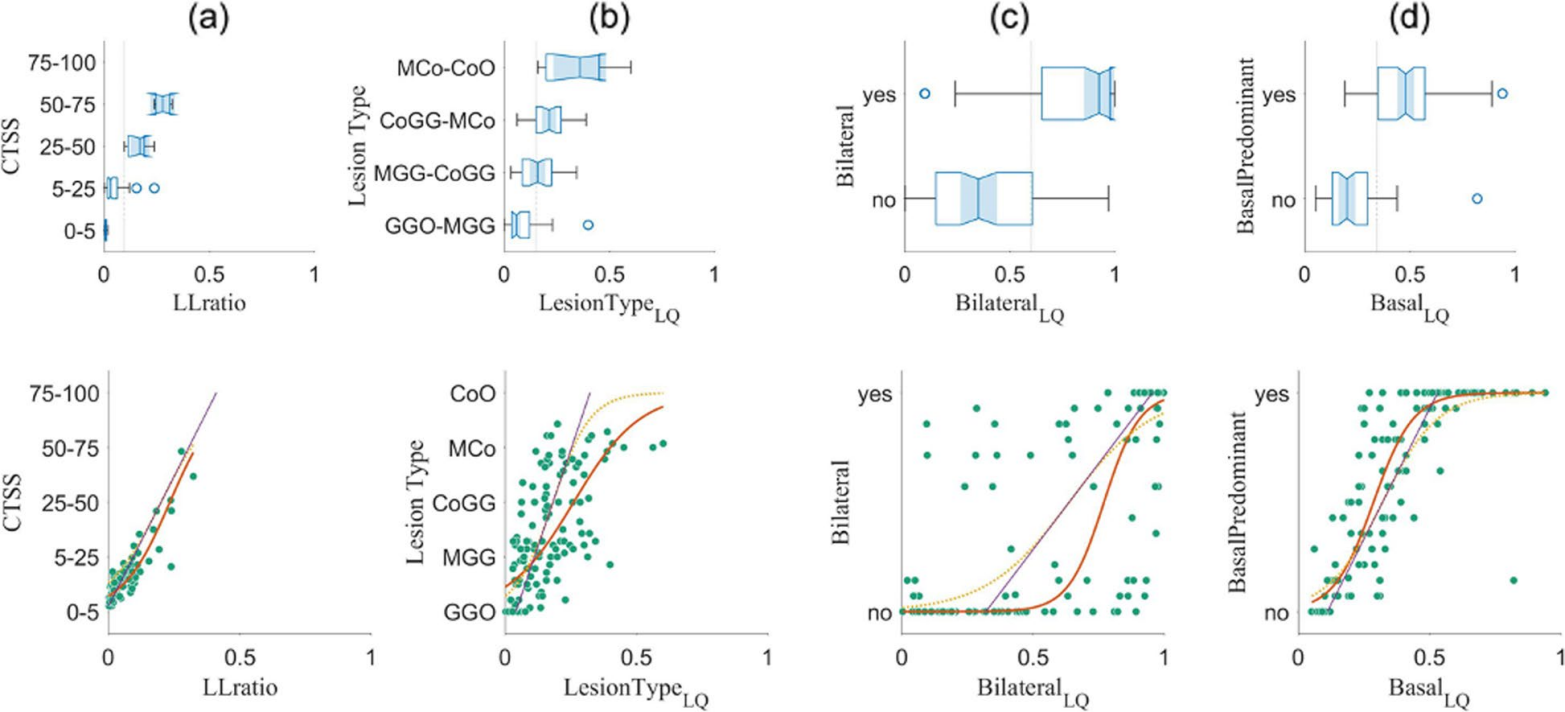
Upper row: distribution of the 120 cases over the four clinical metrics: computed tomography severity score (**a**), lesion type (**b**), bilateral (**c**), and basal predominant (**d**) lesion distribution. On the *x*-axis, there is the quantitative *LungQuant* output corresponding to the qualitative indicator; on the *y*-axis, the visual assessment averaged over all radiologists. For the lesion distribution (bilateral and basal predominant), the grouping is according to the majority of radiologists sharing the same opinion. Youden’s cutoff is shown as a dotted vertical line. Lower row: scatterplot of the average clinical metric of the 120 cases (green dots) *versus* the respective *LungQuant* output. On the same plot, the linear (purple line), linear-constrained sigmoid (yellow line) and the unconstrained sigmoid fit (red line) are shown

We first evaluate the AUC and cutoff for each clinical metric *versus* the corresponding *LungQuant* outputs, computed on the 120 scans and using the information of all radiologists (Fig. 7 top row and Table 4). The proposed dichotomization of the clinical metrics is a “natural” choice given the available classes and can also be compared to the sigmoid inflection point. The *LungQuant*-equivalent clinical metrics all show high AUC with respect to the corresponding clinical values, the latter being averaged on all radiologists (that is, *versus* the estimate of the “true” value).

**Table 4:** AUC, Youden’s cutoff, and sigmoid-fit inflection point on the *LungQuant* outputs *versus* the respective dichotomized clinical metrics

Metric	Strata	*LungQuant* cutoff	AUC	Inflection [95% CL] linear constrained	Inflection [95% CL] unconstrained
CTSS	All	0.10	0.98	0.20 [0.19 0.21]	0.23 [0.23 0.24]
	*>* 400 COVID-19 cases	0.06	0.95	0.17 [0.16 0.18]	0.23 [0.22 0.25]
	*>* 10 years experience	0.09	0.97	0.20 [0.20 0.21]	0.25 [0.23 0.27]
Bilateral	All	0.60	0.85	0.64 [0.52 0.76]	0.77 [0.76 0.78]
	*>* 400 COVID-19 cases	0.60	0.89	0.64 [0.53 0.76]	0.87 [0.87 0.87]
	*>* 10 years experience	0.60	0.85	0.63 [0.50 0.75]	0.67 [0.66 0.68]
Basal predominant	All	0.34	0.90	0.32 [0.31 0.33]	0.29 [0.28 0.29]
	*>* 400 COVID-19 cases	0.32	0.89	0.33 [0.32 0.34]	0.29 [0.29 0.30]
	*>* 10 years experience	0.34	0.91	0.31 [0.30 0.33]	0.28 [0.27 0.28]
Type	All	0.15	0.81	0.18 [0.12 0.25]	0.26 [0.23 0.29]
	*>* 400 COVID-19 cases	0.16	0.80	0.19 [0.12 0.25]	0.29 [0.25 0.33]
	*>* 10 years experience	0.15	0.73	0.16 [0.09 0.22]	0.22 [0.19 0.25]

*AUC* Area under the curve at receiver operating characteristics analysis, *CL* Confidence limits, *CTSS* Computed tomography severity score, *COVID-19* Coronavirus disease 2019

Comparison with radiologists’ stratification based on experience (either COVID-19 related or years of thoracic experience) shows that there was no gain in restricting the evaluators to the experienced ones only. Except for CTSS (where the sigmoid model should not actually apply as we expect a simple linear relationship) and the bilateral assessment, although restricted to the unconstrained sigmoid only, the stratification on experience did not significantly impact the cutoff as well.

The affinity of the inflection point value of sigmoid regression with the AUC cutoff favors the linearly constrained fit. However, if we compare the base parameters (inflection and slope) of the linearly constrained and the unconstrained runs, we see that these are almost always incompatible. Notable exceptions to this trend are the CTSS and the basal predominant sigmoid slopes, for which the relationship between the clinical and the *LungQuant* counterpart is much clearer.

### Image quality

The CT image quality was visually estimated based on individual experience and without formal definition or a priori shared protocol; as such, one should not expect a high level of agreement. The quality metric has the lowest Cohen’s κ (0*.*19 *±* 0*.*13), the second lowest being the κ of the lesion type (0*.*30 *±* 0*.*09). Even with these restrictions, we accounted for the image quality by selecting only CT scans that were not labeled as “low” by any reader. Only 83 scans were selected (ICC values in Table 3). Although some ICC values show some apparent improvement, only the bilateral and basal predominant lesion distribution showed significant improvement when comparing the off-diagonal accuracy values (one-tail *t* test, *p <* 0.001 and *p* = 0*.*027, respectively).

## Discussion

We presented a comparison between a fully automatic, DL-based software and a set of clinical evaluations on some metrics relevant to the management of patients affected by COVID-19 pneumonia. These evaluations were given independently on a set of 120 lung CT scans from a public database and each metric was compared to a simple algebraic manipulation of the software output.

The relevance of the present study consists of three aspects. First, the gathering of qualitative clinical evaluations from a large number of independent radiologists working in their actual clinical settings, according to each own experience, *i.e.*, without consensus and/or shared evaluation protocol. Second, the translation of qualitative assessments into quantifiable metrics, for which the arithmetic mean is the estimator of the “true” (*i.e.*, expected) value. Third, a quantitative characterization using software segmentation outputs, linking them with qualitative evaluations through a simple model.

Even though other authors addressed the present topic, to our knowledge, the only other work in the literature that makes a comparison between the qualitative chest CT parameters obtained visually and using a software in patients with COVID-19 is the report by Colombi et al. [15]. However, the aim of their comparison is not to validate the reliability of the software, but to establish which approach is most informative in predicting specific mortality in COVID-19 patients. Furthermore, results are not directly comparable because the qualitative parameters of chest CT are not the same as those analyzed in our study.

Other works in literature focused on DL systems applied to chest CT scan in COVID-19 pneumonia are [16] and [28]. In [16], authors also compared the performance of an automated DL-based method to manual lung involvement scoring. However, their main aim is to find an optimal threshold for quantification of COVID-involved lung in chest CT and it is different from our aim. In fact, they only consider the CTSS and no other qualitative parameters for the comparison. Nevertheless, their conclusion underlines the importance of the clinical implementation of fully automated methods, thus demonstrating the relevance of our study. In [28], there is not a direct comparison between the output of a DL-based quantification software and the visual evaluation made by radiologists on the CT quantitative parameters for COVID-19 patients, as in our study. They mostly evaluate if quantitative Chest CT could provide reliable information in discriminating COVID-19 from non-COVID-19 patients. Another strength and novelty of our study is in the involvement of 14 clinical experts from 5 clinical centers who visually evaluated the CT scans and provided the ground truth. In [15], two radiologists performed the visual interpretation; in [16], lung involvement was scored by three experienced radiologists; in [28], two radiologists revised the software analysis.

We note that

- The software was not trained to give qualitative characteristics, they were derived a-priori from the segmentation output applying the clinical definition of the metrics (they were not “adapted” to the clinical results);
- The software and radiologists were not informed of any patient’s information on the scans; (c) the number and type of clinical metrics to report was decided by consensus prior to this study, but no formal definition nor analysis protocol was shared; the four clinical metrics were considered to be within the standard practice in the evaluation of COVID-19 cases [23, 29–31].

The radiologist evaluations exhibited a higher heterogeneity than expected, particularly in CTSS and lesion type metrics. Possible explanatory hypotheses can be: the skewed distribution in CTSS (strong prevalence of cases with CTSS 1); the non-uniform utilization of visualization software (it was left to the single radiologist as a personal choice) due to the image format (NIfTI) that forced the use of a visualization tool deemed unusual by some readers; and the multi-center provenance of the scans. The lack of consensus references and shared evaluation protocols is also a probable explanation, although the selected clinical metrics were deemed as common practice and were well known to the participants. Furthermore, since the radiologists’ experience or the perceived quality of the scan did not affect the results, we are inclined to attribute the observed heterogeneity to issues in the visualization procedure due to the utilization of the NIfTI format.

In particular, we underline that the results on the cumulative fraction of cases with *accord* 0.5 on the lesion type indicates that the presence or absence of more severe lesions (Consolidations only) is more consistently evaluated by the radiologist than GG-type lesions (GGO and MGG). In fact, in the case of CoO, the high accord is mainly due to the shared opinion of the readers about the absence of consolidations.

Regarding the image quality and its role, we observe that quality measures could also be operationalized either by providing visual references to participants or by computing objective measures such as the signal-to-noise ratio or the detectability index [32]. These indexes could play a role in the clinical assessment [33], but in this study we believe the subjective evaluation to be more responsive to the question, *i.e.*, whether a scan is eligible for clinical assessment. Scans considered of “low quality”—regardless of their objective noise levels—represent a potential obstacle for the clinician, impairing confidence in their evaluation. This explains why the agreement on quality was so low and why we discarded scans labeled “low” even by a single clinician in the stratification. However, although some improvement was observed because of this restriction, this did not significantly affect the most heterogeneous metrics (CTSS and type of lesion).

Although we believe the sigmoid model to be appropriate to link the *LungQuant-*derived values to the clinical metrics, it is apparent that the regression is impaired in cases where the full range of evaluations is not adequately represented. Since we had very few severe cases in the dataset, the lesion type and the CTSS were substantially biased and the fitted sigmoid parameters should not be taken as final values.

A limitation of this study is the use of a public dataset of CT scans designed for research purposes. Unfortunately, most relevant characteristics (such as scanner type, acquisition parameters, and patient metadata) were not available and images were codified in NIfTI, *Neuroimaging Informatics Technology Initiative*, format, resulting in the additional variability of non-standard visualization tools and departure from the typical clinical workflow on DICOM, *Digital Imaging and Communications in Medicine*, format images. Moreover, in this public database, it is not possible to identify the timing of the scan and it is not specified how the data fit with the clinical outcome of the patient.

The number of scans was chosen to balance statistical aspects and the workload on the clinicians, but the sampling was done on an already imbalanced dataset, particularly in terms of class representation for CTSS. Consequently, some of the results might be impaired, and further efforts are needed to properly validate the regression model.

Although intended, the lack of shared protocols and evaluation standards is a limitation and a second study with operationalized procedures and perhaps a consensus round after the independent evaluation could clarify some of the heterogeneities observed in this work. However, our work could represent a first step to a more robust and larger study.

Despite the heterogeneity of the clinical evaluations, the AUC values are quite satisfactory, and the software is able to distinguish with acceptable precision between the dichotomized clinical metrics. This, together with the clear link between the average clinical metrics and the corresponding software output, suggests that a quantitative aid with automatic software can play a role in improving the clinical workflow related to COVID-19 patients. Precisely because of the heterogeneity of the evaluations, we surmise that the complementary use of quantification software in clinical workflows can fill the support role, *i.e.*, the availability of a software calibrated to the estimated average of a large number of radiologists could provide the necessary evaluation contrast in the interpretation of borderline cases.

## Supplementary Information

**Additional file 1: Supplementary Material 1.** Technical details of the *LungQuant* software. **Supplementary Figure 1. ***LungQuant* analysis pipeline: the first CNN (BBnet) is devoted to the identification of a bounding box enclosing the lungs, performed through a regression. Once the CT scans have been cropped at the bounding boxes, they are used as inputs of both the other CNNs. The second CNN is a U-net trained to segment the lungs with data that contains lung reference masks. The last CNN is a U-net trained to segment the infection, including both Ground Glass Opacities and consolidations. **Supplementary Material 2.** Statistical metrics stratified by readers’ experience and image quality. **Supplementary Figure 2.** Top row: accuracy matrix on the four clinical metrics: CTSS (a), lesion type (b), bilateral (c), and basal predominant (d) lesion distribution. Bottom row: Number of cases per reader and metric class. **Supplementary Figure 3.** Top row: accuracy matrix on the four clinical metrics: CTSS (a), lesion type (b), bilateral (c), and basal predominant (d) lesion distribution. Bottom row: Number of cases per reader and metric class. **Supplementary Figure 4.** Top row: accuracy matrix on the four clinical metrics: CTSS (a), lesion type (b), bilateral (c), and basal predominant (d) lesion distribution. Bottom row: Number of cases per reader and metric class. **Supplementary Figure 5.** Top row: accuracy matrix on the four clinical metrics: CTSS (a), lesion type (b), bilateral (c), and basal predominant (d) lesion distribution. Bottom row: Number of cases per reader and metric class.

## Data Availability

The data used in this study are publicly available at the following link: https://wiki.cancerimagingarchive.net/display/Public/CT+Images+in+COVID-19.

## Abbreviations

AUC: Area under the curve
CoGG: Consolidation and ground-glass
CoO: Consolidations only
CT: Computed tomography
CTSS: CT Severity score
DL: Deep learning
GG: Ground-glass
GGO: Ground-glass only
ICC: Intraclass correlation coefficient
LQ: LungQuant
MCo: Mainly consolidations
MGG: Mainly ground-glass
