# Supplementary material for: A multicenter evaluation of a deep learning software (LungQuant) for lung parenchyma characterization in COVID-19 pneumonia
Source: Eur Radiol Exp. 2023 Apr 10;7:18. doi: 10.1186/s41747-023-00334-z (PMC10083148; doi:10.1186/s41747-023-00334-z)
Supplement: Supplementary file 1 — Additional file 1: Supplementary Material 1. Technical details of the LungQuant software. Supplementary Figure 1. LungQuant analysis pipeline: the first CNN (BBnet) is devoted to the identification of a bounding box enclosing the lungs, performed through a regression. Once the CT scans have been cropped at the bounding boxes, they are used as inputs of both the other CNNs. The second CNN is a U-net trained to segment the lungs with data that contains lung reference masks. The last CNN is a U-net trained to segment the infection, including both Ground Glass Opacities and consolidations. Supplementary Material 2. Statistical metrics stratified by readers’ experience and image quality. Supplementary Figure 2. Top row: accuracy matrix on the four clinical metrics: CTSS (a), lesion type (b), bilateral (c), and basal predominant (d) lesion distribution. Bottom row: Number of cases per reader and metric class. Supplementary Figure 3. Top row: accuracy matrix on the four clinical metrics: CTSS (a), lesion type (b), bilateral (c), and basal predominant (d) lesion distribution. Bottom row: Number of cases per reader and metric class. Supplementary Figure 4. Top row: accuracy matrix on the four clinical metrics: CTSS (a), lesion type (b), bilateral (c), and basal predominant (d) lesion distribution. Bottom row: Number of cases per reader and metric class. Supplementary Figure 5. Top row: accuracy matrix on the four clinical metrics: CTSS (a), lesion type (b), bilateral (c), and basal predominant (d) lesion distribution. Bottom row: Number of cases per reader and metric class. [file 41747_2023_334_MOESM1_ESM.pdf]

ELECTRONIC SUPPLEMENTARY MATERIAL

A multicenter evaluation of a deep learning software (LungQuant) for lung parenchyma characterization in COVID-19 pneumonia

Supplementary Material 0.1 - Technical details of the *LungQuant* software

The last version of the *LungQuant* software is an updated version of [1]. The main difference is the addition of the first of the three neural networks in a cascade structure. It is a CNN devoted to the identification of a bounding box enclosing the lungs, performed through a regression. Once the CT scans have been cropped at the bounding boxes, they are used as inputs of both the other CNNs for segmentation. It has been added to make the system work also on CT images acquired with a different Field Of View. This final updated pipeline is represented in Supplementary Figure 1. Another upgrade in the software functionality, with respect to the first version described in [1], was the introduction of a function that separates right and left lungs with two different masks, and a threshold to differentiate consolidations from GGO in the lesion mask.

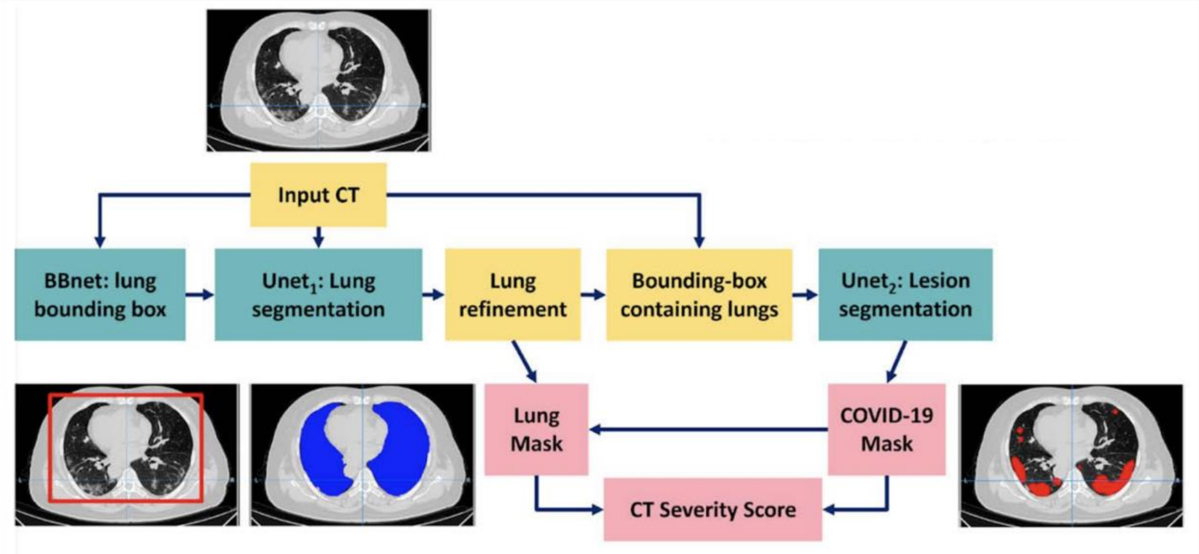

Supplementary Figure 1. *LungQuant* analysis pipeline: the first CNN (BBnet) is devoted to the identification of a bounding box enclosing the lungs, performed through a regression. Once the CT scans have been cropped at the bounding boxes, they are used as inputs of both the other CNNs. The second CNN is a U-net trained to segment the lungs with data that contains lung reference masks. The last CNN is a U-net trained to segment the infection, including both Ground Glass Opacities and consolidations.

This updated version of the algorithm underwent the same training procedure as the first version [1], and the validation was made through a test on the same benchmark dataset [2], which is an independent set of images not used in the training phase.

After the training phase, the metrics used to validate the segmentation performance were surface and volumetric Dice similarity coefficients (sDSC and vDSC), computed on the independent test dataset [2], which is the only one that contains both lung and lesion reference masks. The sDSC at 5 mm of tolerance (sDSC 5mm) and the vDSC for lung segmentation obtained on the test set, regarding the last version of the software, are equal to 0.97 0.01 and 0.96 0.01, respectively. For the lesion segmentation, the performances in terms of sDSC 5mm and the vDSC are equal to 0.83 0.07, and 0.69 0.08, respectively. The system was also evaluated in terms of Mean Absolute Error (MAE). The MAE in assessing the percentage of infected lung volume is equal to 2%. Another metric used to evaluate the automatic system is the accuracy in assigning the correct CT-SS class, which is 80%.

Supplementary Material 0.2 - Statistical metrics stratified by readers' experience and image quality

The analysis of readers' concordance described in Section 2.6.1 has been repeated stratifying by the readers' expertise in reading CT scans of COVID-19 patients and by their assessment of image quality. This means first, the analysis has been restricted to the opinions of the readers with specific expertise, quantified as the number of CT COVID-19 cases evaluated in their experience. Then, the analysis has been repeated accounting for the image quality, i.e., by selecting only those CT scans which were consistently labelled "*acceptable*" or "*optimal"*. With this stratification, the analysis is restricted to only 83 scans.

In Supplementary Figure 2 accuracy values are given only for the three more expert readers who have evaluated more than 400 CT COVID-19 cases in their experience. For the same three readers, in the same figure, we report the number of cases per lesion class and reader. The agreement is shown for all the clinical metrics (CTSS, Lesion type, Bilateral and Basal Predominant lesion distribution).

In Supplementary Figure 3 accuracy values are given only for the less expert readers who have evaluated less than 100 CT COVID-19 cases in their experience. For the same three readers, in the same figure, we report the number of cases per lesion class and reader. The agreement is shown for all the clinical metrics (CTSS, Lesion type, Bilateral and Basal Predominant lesion distribution).

In Supplementary Figure 4 accuracy values are given only for the less expert readers who have evaluated less than 100 CT COVID-19 cases in their experience. For the same three readers, in the

same figure, we report the number of cases per lesion class and reader. The agreement is shown for all the clinical metrics (CTSS, Lesion type, Bilateral and Basal Predominant lesion distribution).

In Supplementary Figure 5 accuracy values are given for all 14 readers but considering only the 83 scans consistently labelled with an “*acceptable*” or “*optimal”* image quality. For these 83 selected scans, in the same figure, we report the number of cases per lesion class and reader. The agreement is shown for all the clinical metrics (CTSS, Lesion type, Bilateral and Basal Predominant lesion distribution).

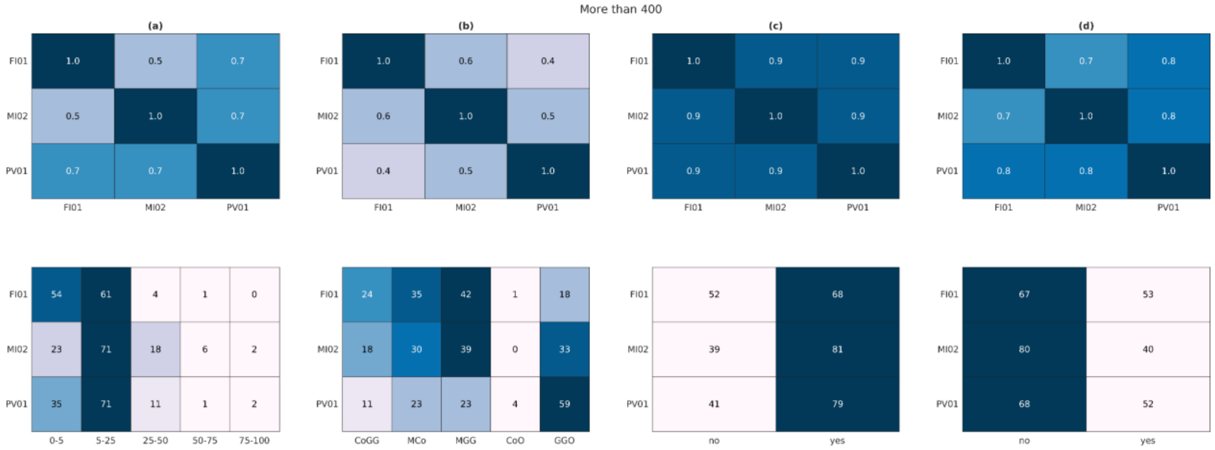

Supplementary Figure 2. Top row: accuracy matrix on the four clinical metrics: CTSS (a), lesion type (b), bilateral (c), and basal predominant (d) lesion distribution. Bottom row: Number of cases per reader and metric class.

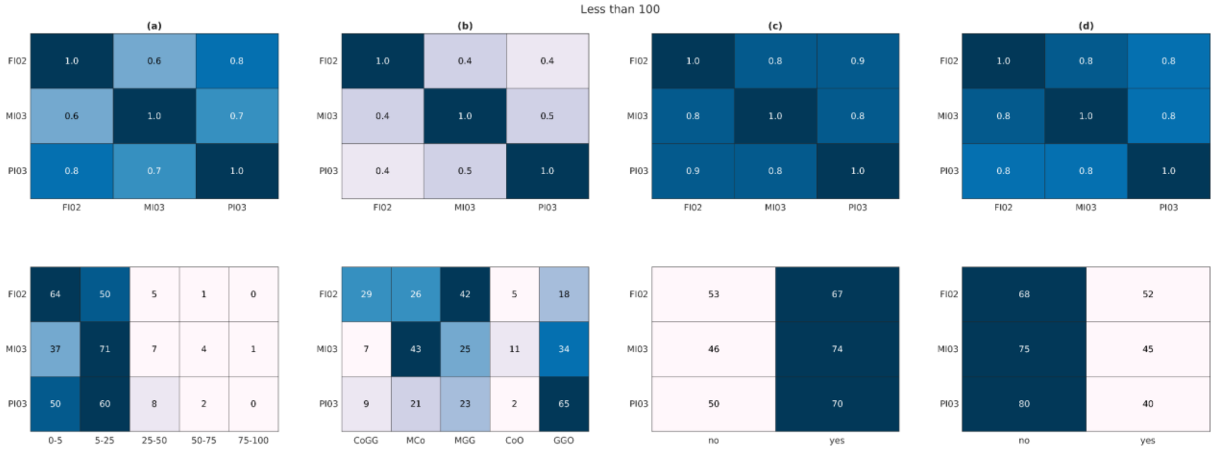

Supplementary Figure 3. Top row: accuracy matrix on the four clinical metrics: CTSS (a), lesion type (b), bilateral (c), and basal predominant (d) lesion distribution. Bottom row: Number of cases per reader and metric class.

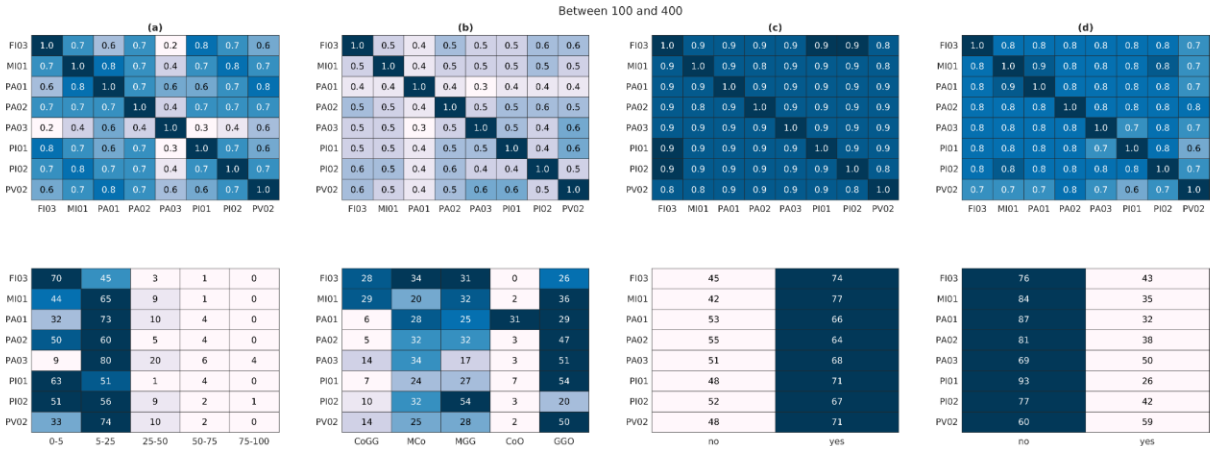

Supplementary Figure 4. Top row: accuracy matrix on the four clinical metrics: CTSS (a), lesion type (b), bilateral (c), and basal predominant (d) lesion distribution. Bottom row: Number of cases per reader and metric class.

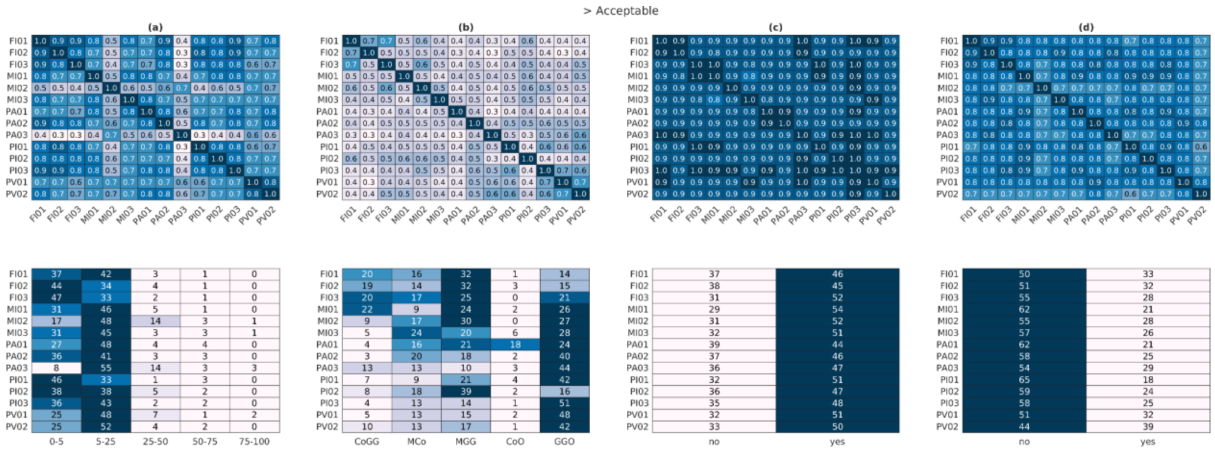

Supplementary Figure 5. Top row: accuracy matrix on the four clinical metrics: CTSS (a), lesion type (b), bilateral (c), and basal predominant (d) lesion distribution. Bottom row: Number of cases per reader and metric class.

References

[1] F. Lizzi, A. Agosti, F. Brero, R. F. Cabini, M. E. Fantacci, S. Figini, A. Lascialfari, F. Laruina, P. Oliva, S. Piffer, et al., Quantification of pulmonary involvement in covid-19 pneumonia by means of a cascade of two u-nets: Training and assessment on multiple datasets using different annotation criteria, International journal of computer assisted radiology and surgery 17 (2) (2022) 229–237.

[2] COVID-19-CT-Seg dataset, https://zenodo.org/record/3757476#.YoOChjYzbeo, [Accessed: 2022-06-22].
